# Probabilistic Daily ILI Syndromic Surveillance with a Spatio-Temporal Bayesian Hierarchical Model

**DOI:** 10.1371/journal.pone.0011626

**Published:** 2010-07-16

**Authors:** Ta-Chien Chan, Chwan-Chuen King, Muh-Yong Yen, Po-Huang Chiang, Chao-Sheng Huang, Chuhsing K. Hsiao

**Affiliations:** 1 Institute of Epidemiology, College of Public Health, National Taiwan University, Taipei, Taiwan; 2 Division of Health Policy Research, Institute of Population Health Science, National Health Research Institutes, Zhunan, Taiwan; 3 Department of Public Health, College of Public Health, National Taiwan University, Taipei, Taiwan; 4 Department of Disease Control and Prevention, Taipei City Hospital, Taipei City Government, Taipei, Taiwan; 5 Graduate Institute of Biomedical Informatics, Taipei Medical University, Taipei, Taiwan; University of East Piedmont, Italy

## Abstract

**Background:**

For daily syndromic surveillance to be effective, an efficient and sensible algorithm would be expected to detect aberrations in influenza illness, and alert public health workers prior to any impending epidemic. This detection or alert surely contains uncertainty, and thus should be evaluated with a proper probabilistic measure. However, traditional monitoring mechanisms simply provide a binary alert, failing to adequately address this uncertainty.

**Methods and Findings:**

Based on the Bayesian posterior probability of influenza-like illness (ILI) visits, the intensity of outbreak can be directly assessed. The numbers of daily emergency room ILI visits at five community hospitals in Taipei City during 2006–2007 were collected and fitted with a Bayesian hierarchical model containing meteorological factors such as temperature and vapor pressure, spatial interaction with conditional autoregressive structure, weekend and holiday effects, seasonality factors, and previous ILI visits. The proposed algorithm recommends an alert for action if the posterior probability is larger than 70%. External data from January to February of 2008 were retained for validation. The decision rule detects successfully the peak in the validation period. When comparing the posterior probability evaluation with the modified Cusum method, results show that the proposed method is able to detect the signals 1–2 days prior to the rise of ILI visits.

**Conclusions:**

This Bayesian hierarchical model not only constitutes a dynamic surveillance system but also constructs a stochastic evaluation of the need to call for alert. The monitoring mechanism provides earlier detection as well as a complementary tool for current surveillance programs.

## Introduction

A timely surveillance system is a crucial tool for early prevention of influenza and for urgent intervention when a potentially lethal strain of influenza emerges. Otherwise, this highly contagious infectious disease may spread rapidly in a short period of time as it did in the influenza pandemic of 2009 [1], [2]. Traditionally, routine influenza surveillance [3] adopts voluntary-based sentinel surveillance for evaluation of both vaccination effectiveness [4] and health policy performance [5]. However, possible delays in the self-reporting schedules as well as short incubation periods usually lead to lack of timeliness in detecting influenza aberrations [6]. Another alternative is emergency room surveillance [7], [8], where influenza-like illness (ILI) visits are monitored with counts based on chief complaints or clinical diagnosis using the International Classification of Diseases (ICD) codes. Such ILI based syndromic surveillance for influenza achieves better timeliness than either laboratory surveillance [9] or sentinel surveillance [10].

Although all surveillance algorithms involve aberration detection and decision making, evaluation of the uncertainty inherent in such prediction and the spatial-temporal characteristics involved has been only partially addressed. For examples, commonly employed current methods, such as historical limit [11], Serfling [12], Cusum [12], [13], and exponentially weighted moving average (EWMA) [14], do not address properly the uncertainty assessment in prediction. Less commonly used Bayesian approaches, which do in contrast assess this intrinsic uncertainty, can provide proper stochastic statements that reflect the inference procedure. Examples include Markov models [15], [16], multivariate autoregressive processes [17], dynamic Bayesian models [12], [18], EWMA with non-heterogeneity spatial smoothing [14], Bayesian information fusion networks [19] and Bayesian hierarchical models with meteorological factors [20], which have been adopted in several studies for influenza surveillance. Most of these methods, however, have been applied only with temporal data employing a time unit of a week or longer. Here we propose a hierarchical model for daily ILI visits, which addresses both the timeliness issue and the issue of evaluating the uncertainty inherent in prediction. In addition to temporal variation, spatial patterns and meteorological factors also deserve special attention. In metropolitan areas, patients may have more than one choice available of where to seek medical treatment. Therefore, it is essential to consider the spatial interaction between different areas or hospitals to better explain the observed pattern of ILI visits. Furthermore, the inclusion of the correlated effects among neighboring hospitals or areas may help to model the pattern of influenza spread. Meteorological factors [21] have also been shown to play an influential role with regard to influenza illness and are worth including in the surveillance system. An association between the transmission/survival of influenza virus and meteorological factors such as temperature [22], [23], relative humidity [24], vapor pressure [22], and solar radiation [25] has been observed in animal and ecological studies. In order to enhance prediction accuracy, our proposed model takes all these factors into consideration.

The aim of this study is to construct a timely influenza surveillance system based on a Bayesian hierarchical model which incorporates the impact of spatial and temporal dependence, accounts for meteorological information, and performs probabilistic prediction on influenza activities. Unlike the traditional binary representation for signal alert, the predicted probabilities can imply the strength of possible aberrations for use in further decision making. We will illustrate the proposed approach by considering numbers of ILI visits observed in Taipei City.

## Materials and Methods

### Materials

The daily surveillance data were collected in emergency rooms of five community hospitals in Taipei City from September of 2005 to February of 2008 [7]. The 2005 data were not recorded on a daily basis and hence we considered these three months a grace period. The observations from 2006 to 2007 were taken as the training data to construct the statistical model, while the rest were used for testing it. Influenza-like illness (ILI) syndrome was defined by a composite group of ICD-9 (International Classification of Diseases, ninth revision) codes previously validated by physicians [26]. Daily meteorological data taken during the same period of time, including average temperature and vapor pressure, were accessed through the Taipei station of Taiwan's Central Weather Bureau (TWCWB).

### Spatial Structure and Interaction

**Figure 1:**
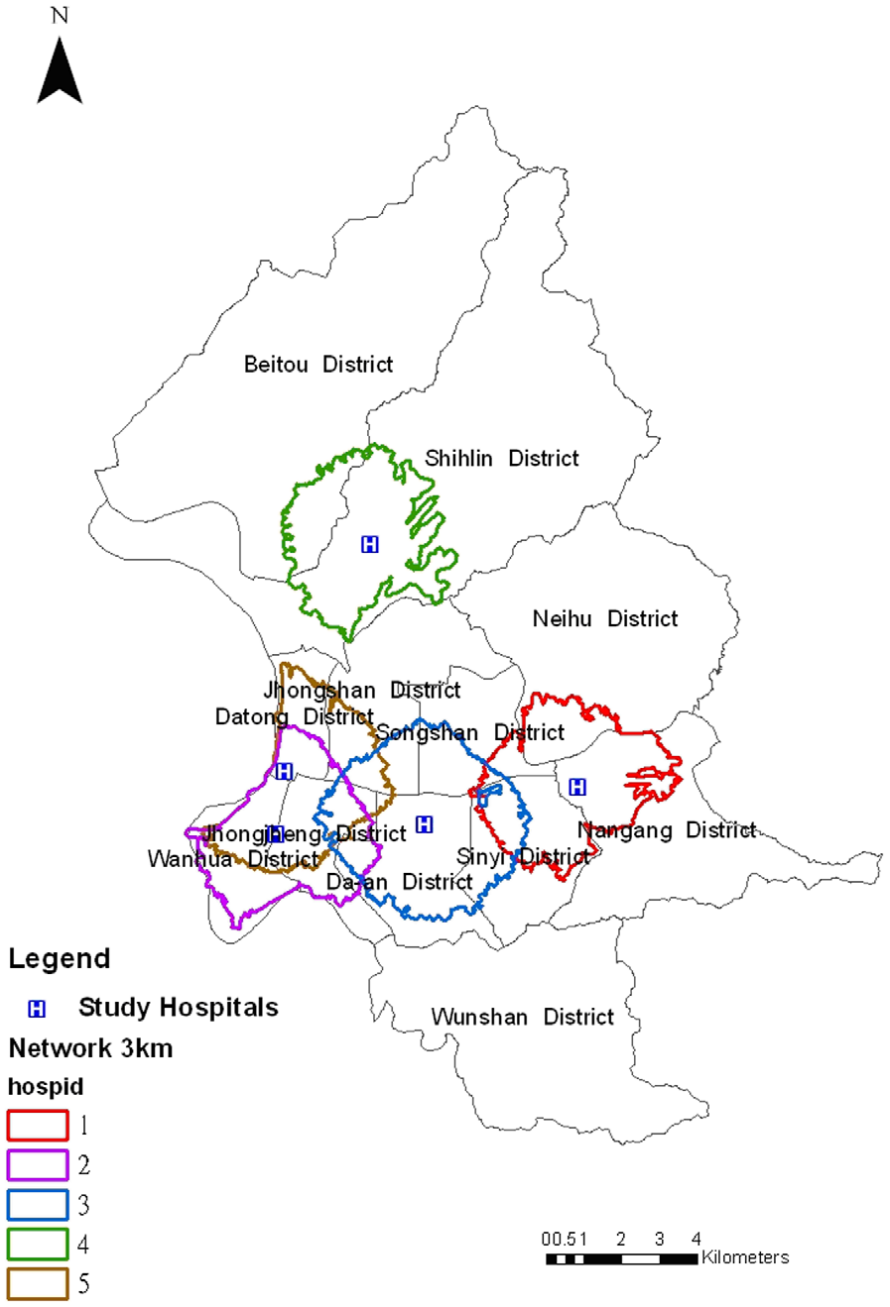
Spatial distribution of the five hospitals and corresponding buffers.

For each hospital, its spatial neighborhood was defined as a buffer of 3 kilometers (Figure 1) calculated by Network extension of ArcGIS (ArcMap, version 9.0; ESRI Inc., Redlands, CA, USA). The choice of 3 kilometers was based on a maximum transportation time of 4.5 minutes from a patient's home to the hospital at the velocity of 40 km/hr without consideration of traffic lights. The population at risk was calculated based on the smallest census unit, *Li* (Figure S1). The residents living within a buffer were considered the only potential visitors of that hospital. For adjacent or overlapping buffers, we assumed the existence of spatial interaction and imposed a spatial structure on the ILI observations collected from the corresponding hospitals, whereby residents living in the buffer areas were assigned equal chance of visiting each hospital.

### Bayesian Model Formulation

Let 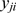 denote the number of ILI visits for the 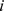-th buffer area on the 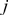-th day, where 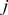 = 1,…, 5 and 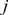 = 1,…, 730 for the model construction stage. For 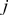 = 731,…, 786, the observations 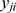 were left out for validation. The daily ILI visits at emergency rooms were assumed to follow a Poisson distribution. In other words, the local variability of the number of ILI visits is
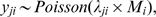
where the mean parameter 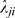 is the expected risk for the corresponding buffer area and time interval, and 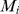 the population at risk in the buffer area.

The logarithm of 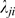 can be next expressed as
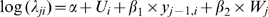


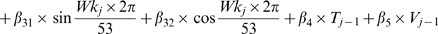
where 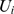 stands for an intrinsic Gaussian conditionally autoregressive distribution,
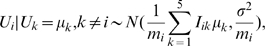
with 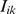 = 1 if areas 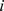 and 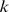 are adjacent or their corresponding buffers overlap, and 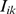 = 0 otherwise. The coefficient 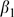 stands for the effect from the previous day 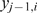, and 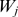 is an indicator variable for weekends, national holidays, and Chinese New Year. The terms 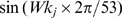 and 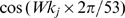 represent the seasonal cycle with 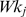 representing the number of weeks in a year for the 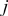-th day, while 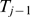 represents the standardized daily average temperature, and 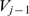 the standardized daily average vapor pressure. The reference values for standardization were taken to be the mean and standard deviation of the daily average temperature and vapor pressure during 2006–2007. For regression coefficients and precision parameters, we adopted reference priors. The complete model specification is described in detail in Model S1.

### Posterior Samples for Inference

All computations were based on posterior samples derived via Markov chain Monte Carlo (MCMC) methods with the free statistics software WinBUGS 1.4.3 [27]. Three chains were run with 10,000 iterations each. The first 5,000 iterations of every chain were discarded in order to remove the possible dependence on initial values, and the value of the sampling interval (thinning value) was 5. This resulted in 15,000 posterior samples for each parameter. We next applied the “Coda” library of the R statistical package [28] to perform the convergence test with the Gelman and Rubin statistic [29], where the statistic was checked if smaller than 1.1 with an effective sample size larger than 200 for all parameters. Various specifications of prior distributions were also considered to examine the sensitivity of the posterior inference.

### Model Validation

We carried out validation analysis with the ILI data observed in the first two months of 2008 based on the prediction model trained by the observations collected in the previous 730 days. This two-month period contained a wide range of “days” including weekdays, weekends, and long holidays. Two types of prediction were performed: one was short-term prediction on a weekly basis, and the other was long-term monthly prediction. The precision was measured by the average prediction error (APE, 
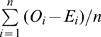
) and the average relative root mean squared error (ARRMSE, 
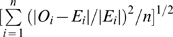
). In addition, we computed the Pearson's correlation to evaluate the consistency between the observed and expected ILI visits, where the daily expected ILI visits were calculated based on estimates of the posterior means 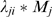.

### Probability of Alerts

For risk prediction on any day 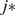 at hospital 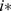, we denote 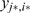 as the number of ILI visits and consider the posterior probability of 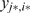 exceeding the threshold,
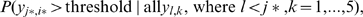
as a measure of the need to call for alert. Note that this posterior probability is a conditional probability conditioning on all observations 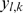 for 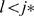 from all hospitals 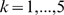, including 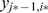 which appears in the log-link regression function. The use of probabilities for risk estimation provides a natural and intuitive assessment for uncertainty. Here we adopted a dynamic threshold, which was the maximum expected ILI visits of the previous 7 days, and utilized the posterior probability of 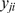 exceeding the threshold as a measure of need for alert. If this posterior probability is large, it indicates a high chance of influenza epidemic and, therefore, public health authorities may opt to initiate a pandemic preparedness plan. If it is small, then most likely the chance of an influenza outbreak is slim and no additional action needs be taken. The choice of such threshold is able to capture the dynamic trend of ILI observations because each estimated ILI visits is derived through a posterior probability condition on all previous observations and particularly on the ILI counts in the previous day through regression modeling. In other words, if the number of ILI cases is on the rise during the epidemic season, the maximum of the ILI visits among the previous seven days will increase and thus the threshold will elevate synchronously.

The WinBUGS 1.4.3 [27] and R [30] source codes for our Bayesian approach, as well as the implemented predictions and graphs, are available in Methods S1 and Methods S2 (more detailed information can be found in the website http://homepage.ntu.edu.tw/~ckhsiao/download.html). We compare the proposed method with the influenza virus isolation rate collected by Taiwan CDC [31] for the whole of Taiwan, and with the modified Cusum methods [13] with the R codes described in Watkins et al. [32].

## Results

### Model Training

**Figure 2:**
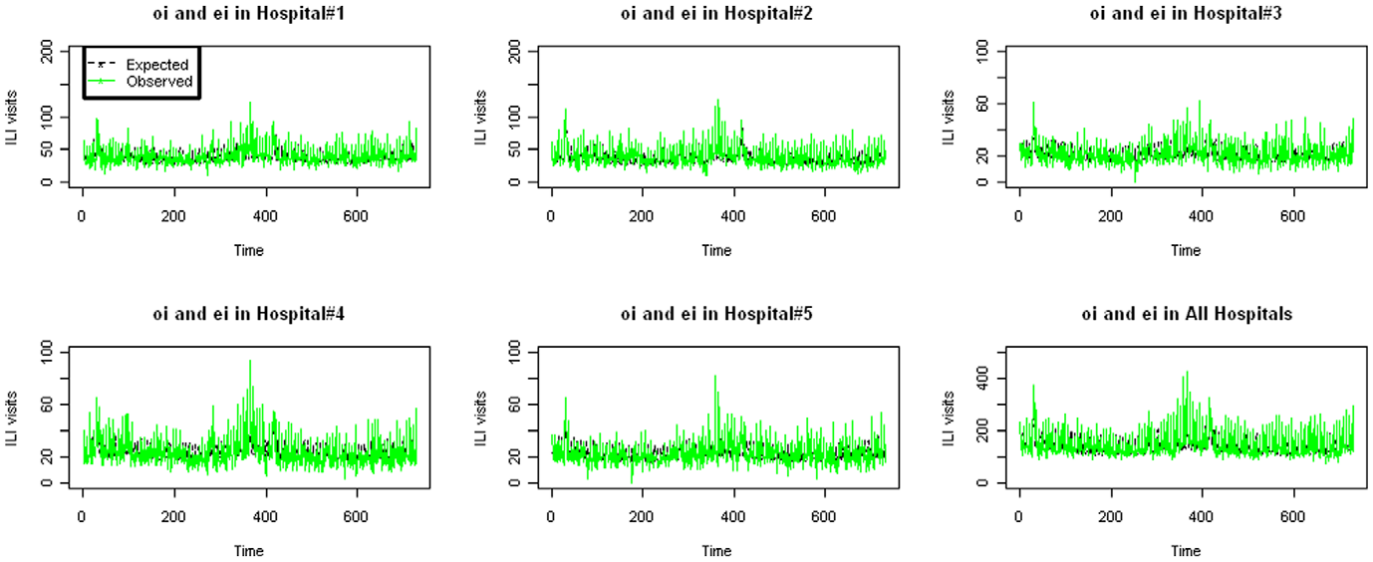
Temporal patterns of observed (oi) and expected ILI (ei) visits during 2006–2007.

The descriptive statistics for daily ILI visits and for meteorological factors during 2006–2007 are shown in Table 1 (frequency distributions are shown in Figure S2). In general, Hospital 1 (H1) and Hospital 2 (H2) had higher ILI visits than other hospitals (p<0.001). After convergence diagnosis, the final model was constructed and details of the model parameters are contained in Table S1. The observed and expected ILI visits show similar patterns with a high correlation (Pearson's correlation r = 0.8, p<0.0001). Figure 2 shows a consistent trend between these two measures for ILI visits in each hospital as well as for overall ILI visits. The overall average residual of prediction is 0.34, and the average relative root mean squared error is 0.17 (Table 2).

**Table 1:** Descriptive statistics for daily ILI visits in five hospitals (H1–H5), and for meteorological factors during 2006–2007.

Variables	N (Days)	Min.	Max.	Mean	Median	Std. Deviation
ILI visits in H1	730	11	122	40.38	37	13.92
ILI visits in H2	730	10	127	38.37	35	15.62
ILI visits in H3	730	0	62	21.64	20	8.08
ILI visits in H4	730	3	94	23.33	21	10.84
ILI visits in H5	730	0	82	22.92	21	8.99
ILI visits in All Hospitals	730	76	430	146.64	131	49.28
Average temperature (°C)	730	9.8	32.4	23.70	24.1	4.98
Vapor pressure (hPa)	730	7.9	33	22.79	22.5	6.12

**Table 2:** Prediction accuracy for each hospital and for all 5 hospitals.

Hospital	APE	ARRMSE	Correlation
H1	0.38	0.24	0.69*
H2	0.37	0.27	0.72*
H3	0.21	0.27	0.66*
H4	−0.82	0.30	0.69*
H5	0.20	0.29	0.64*
All	0.34	0.17	0.8*

APE: Average Prediction Error.

ARRMSE: Average Relative Root Mean Squared Error.

*p-value<0.0001.

**Figure 3:**
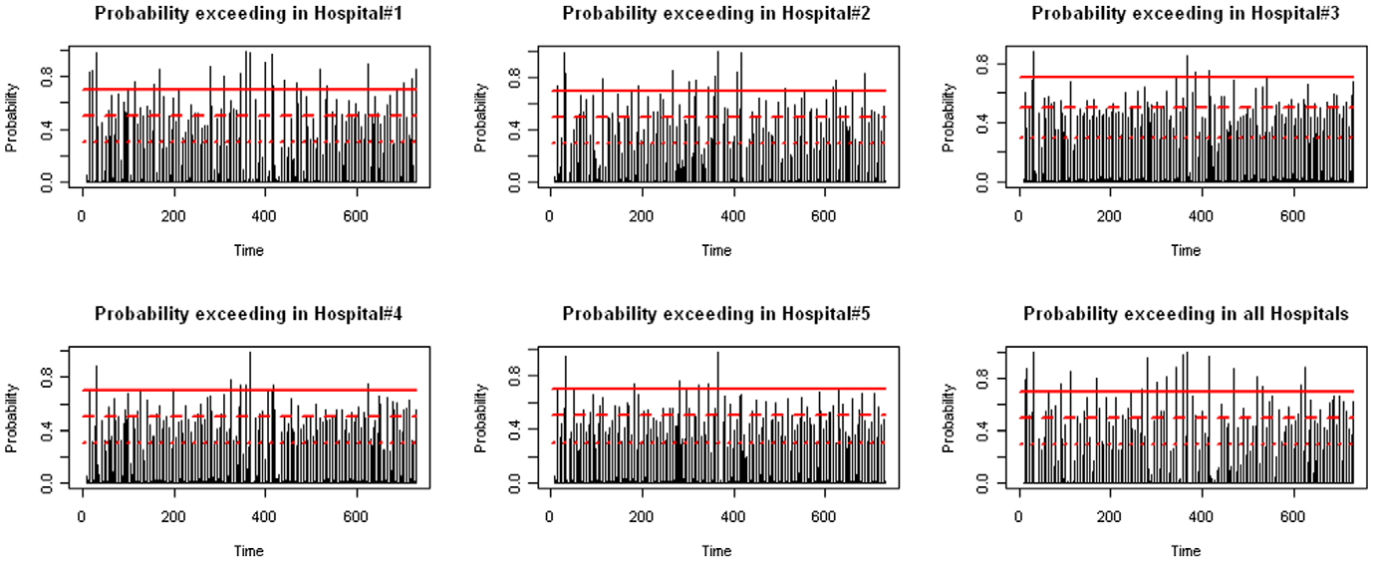
Probability of alert at the stage of model fitting. Top line is for posterior probability = 0.7, middle for 0.5, and bottom for 0.3.

To examine the posterior probabilities of alert, we plot the probabilities in Figure 3. Any value located above the top line (probability = 0.7) indicates a need for alert. Values below the middle line (probability = 0.5) indicates a small chance for outbreak. For most of the days (about 74% to 82%), the probabilities are below the bottom line value of 0.30 (see Table S2 for numbers and percentages of days with posterior probabilities in different ranges), indicating no apparent danger of influenza epidemic during most of the period under consideration. About 1% to 4% of this period the posterior probabilities fall in the highest range (0.71, 1.00), which indicates that these days deserve additional attention for possible outbreak. The average of the corresponding observed ILI visits for these days is indeed 248.83.

### Model Validation

To evaluate the model performance, we use the ILI data from January and February of 2008 for validation. The summary statistics of the daily ILI and meteorological factors are listed in Table 3. Similar to the training data, the numbers of ILI visits in Hospitals 1 and 2 are significantly higher than the other three hospitals (p<0.001). Validation of the model has been conducted by considering two different time scales, weeks and months. For the weekly prediction, the average prediction error is 0.44 and the average relative root mean squared error is 0.33 for this 8-week period (Table 4). For the monthly prediction, the average prediction error is −5.03 and the average relative root mean squared error is 0.37.

**Table 3:** Descriptive statistics for daily ILI visits in five hospitals (H1–H5), and for meteorological factors from January, 2008 to February, 2008.

Variables	N (Days)	Min.	Max.	Mean	Median	Std. Deviation
ILI visits in H1	60	25	98	48.08	42	17.93
ILI visits in H2	60	14	100	41.67	36.5	16.90
ILI visits in H3	60	10	48	22.98	21	8.10
ILI visits in H4	60	8	71	27.58	23	13.27
ILI visits in H5	60	9	54	26.82	24.5	10.07
ILI visits in All H	60	83	319	167.13	143.5	59.66
Average temperature (°C)	60	9.3	22.8	15.36	15.3	3.13
Vapor pressure (hPa)	60	7.6	19.8	14.48	14.65	2.88

**Table 4:** Prediction accuracy for validation with two time scales.

Weekly Prediction				Monthly Prediction			
Days for updating model	Days for validation	APE	ARRMSE	Days for updating model	Days for validation	APE	ARRMSE
1–730	731–737	14.73	0.25	1–730	731–758	3.00	0.40
1–737	738–744	10.39	0.34	1–737	738–765	−6.67	0.43
1–744	745–751	−5.34	0.57	1–744	745–772	−10.65	0.42
1–751	752–758	−10.55	0.38	1–751	752–779	−8.34	0.31
1–758	759–765	−21.39	0.42	1–758	759–786	−2.50	0.30
1–765	766–772	4.52	0.26	-	-	-	-
1–772	773–779	−1.47	0.09	-	-	-	-
1–779	780–786	12.63	0.32	-	-	-	-
Average =		0.44	0.33	Average =		−5.03	0.37

**Figure 4:**
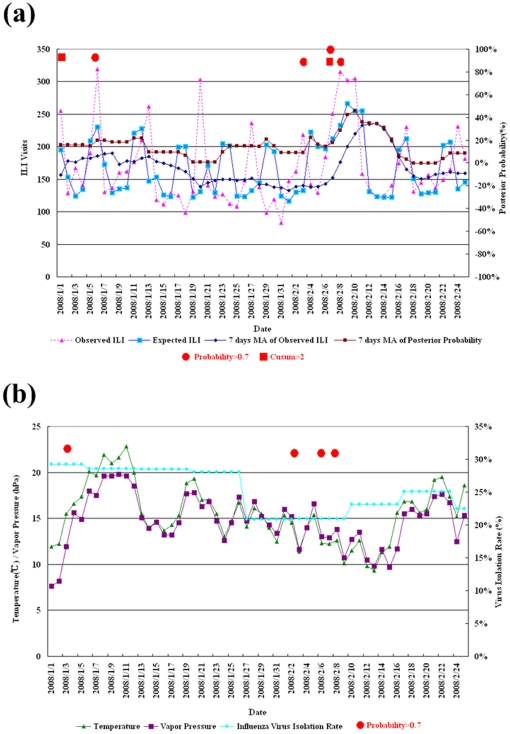
Temporal chart of ILI visits, different alerts and associated factors. (**a**) ILI counts and probability of alert during the validation stage based on weekly updated parameters. (**b**) The time series plots of ILI visits, weekly influenza isolation rate, temperature and vapor pressure, respectively.

Figure 4(a) displays the predicted ILI visits, along with the observed, for the validation period. Both share the same patterns of rises and falls. Particularly, the predicted ILI counts tend to rise 1–2 days earlier than the observed ILI counts, which may be acknowledged as a desirable property for influenza alarm system. In addition, we plot a 7-day simple moving average (MA) for the observed ILI visits to indicate a smoothing trend. Again, the peak around February 10 in the MA plot is successfully detected by the Bayesian predicted counts and by the posterior decision rule. Next, the days with posterior probability of alert larger than 0.7 are indicated with solid circles in the same figure. It shows that there are three days with probability of alert reaching the 70% threshold. These three highest posterior probabilities occur on January 6, February 4, and February 9 in 2008; all in the 2007–08 influenza season which began on October 1, 2007. The largest number of ILI visits, 319, was observed on January 6, 2008, with a corresponding estimated alert probability of 87.83% (Y-axis in the right). On February 3 in 2008, the temperature reached the lowest (11.4°C) in the first wave and the vapor pressure was also low (11.6 hPa). On February 4, 2008, the predicted probability of alert reaches a height of 91.08%, whereas the observed upsurge trend of ILI visits takes place on February 6, the Chinese New Year eve. From February 8 to February 9, both the estimated numbers of ILI visits and the posterior probability of alert reach the highest range. During this validation period, the probability of alert does in fact exceed 70% at the early and middle stage of this influenza epidemic.

To further examine the association between ILI visits, daily average temperature, and vapor pressure, we compare their time series plots in Figure 4(b). The patterns of the daily average temperature and vapor pressure are highly correlated (r = 0.89, p<0.01). The time lag between the drop in temperature (or vapor pressure) and the rise in ILI is around 2 days, indicating strong support for their relation. Another informative indicator would normally be the influenza isolation rate, which was stable at 20% before the Chinese New Year. However, the long holiday effect prevents the collection of specimens, and therefore the sharp decrease in isolation rate fails to reflect the true epidemic trend during the Chinese New Year holidays.

### Comparison with Cusum Method

To compare our procedure with the modified Cusum method, we employ the validation data as well. Data from January 1–8 of 2006 are used for baseline calculation, and the next 778 days (from January 9, 2006 to February 25, 2008) are included for Cusum evaluation. The daily signals under the modified Cusum signals with the decision interval set at 2 are shown in Figure 4(a) alongside our proposed probability of alert. The Cusum method detects two signals, one on 1/1 and the other on 2/8. The latter is a successful hit, while the former seem a less emergent case. The Cusum and our proposed approach provide similar signals. For instance, in the training data, the numbers of days whose posterior probability of alert falls in the four ranges (0–30%, 31–50%, 51–70%, 71–100%) are 639, 60, 49, and 30 days, respectively; while the average of the corresponding Cusum values in each group are 0.09, 0.71, 0.94, and 1.12, respectively. There are 21 days where the Cusum values are larger than 2, and these 21 days correspond to an average posterior probability of 0.59.

## Discussion

This paper presents a Bayesian hierarchical model, with both spatial and temporal structures simultaneously, to construct a probabilistic measure for the likelihood of an outbreak of influenza epidemic. This model provides the strength of information for epidemic alert and captures the uncertainty inherent in prediction. A further advantage of this approach is its inclusion of (a) real road networks between community hospitals as the intrinsic correlation spatially, (b) buffer construction for hospital service areas, and (c) effects of meteorological factors. This model can be easily extended to incorporate other covariates or appropriately modified with better suited definitions of buffers for different cities and countries.

For prediction and alert purposes, the posterior probability of 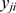, the number of ILI visits in the future 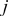
–th day at the 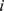
–th hospital, exceeding the threshold is reported. This threshold is chosen to be the maximum number of expected ILI cases in the previous seven days at the same hospital. In other words, this maximum value is associated with estimated 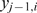, 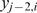, … and 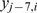, where the estimates are posterior means related to all previously observed ILI counts and specifically to the observation in the day before. The threshold is dynamic. It becomes large if the number of cases is on the rise, and turns small if the epidemic is under control. This choice is able to reflect timely the occurrence of influenza. Another advantage of using such conditional probability and threshold is that it takes into account the seasonality of flu. The terms sine and cosine in the regression model explain only part of the annual trend, but do not account for the effects from specific seasons. However, this seasonality effect will be implicitly inherent in the seven observations 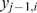, 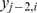, … and 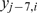, and therefore involves in both the prediction of posterior probability and the value of threshold.

The modified Cusum [13] has been applied in the early aberration reporting system (EARS) of the Centers for Disease Control (CDC) in the United States, which has been used for bioterrorism detection and syndromic surveillance. Three different methods were implemented, called C1, C2 and C3, with different sensitivity levels, whereas this study adopts C3, the one with the highest sensitivity, to compare with our proposed method. The C3 and the posterior probability scheme are complementary in epidemic detection. For instance, C3 detects a large influx of ILI visits at the first day, while our Bayesian model manifests an earlier and stronger signal before the peaks of ILI visits. Their major difference, however, is in the inclusion of explanatory variables. The modified Cusum predicts the trend based on only ILI visits, without considering meteorological factors, seasonality, weekend/holiday effect and spatial interaction between neighboring hospitals.

The incorporated covariates have important contributions and features. First, temperature and vapor pressure have been reported to associate negatively with influenza transmission [22], where the environmental conditions provide the niche factors for survival and transmission of the influenza virus. This can be observed in the lag time between the temperature drop and the rise of the ILI visits. For instance, low temperature and low vapor pressure occurred on February 3 of 2008, causing the probability signal to surge on February 4 to a large magnitude of 91.08%. At this time a public health alert should have been activated. In fact, the observed ILI visits surged on February 6, after the biological incubation time of 2–3 days for influenza infection. The probability of alert a priori and the predicted ILI cases both match the observed pattern well. To incorporate weather factors, users can consider either the weekly forecast or the historical average to carry out the Bayesian prediction of influenza epidemic.

Second, in daily syndromic surveillance, a simple 7–day moving average plot is usually considered to demonstrate the observed pattern and to smooth the weekend effect in data collected at emergency rooms. In Taiwan, most hospitals and clinics do not provide weekend outpatient services [33], and thus patients rush into emergency rooms on weekends. The ratio of ILI visits on weekends to those on weekdays was in fact around 1.45 during our study period. Therefore, we deliberately integrate weekend and holiday effects into the model.

Third, other formulations of spatial heterogeneity for ILI visits among different hospitals could easily be modeled. In this study, the spatial interaction is constructed based on network buffers without considering traffic conditions or other factors. If the hospitals in question are to have different service areas, then this information could be included accordingly. Taipei City is about 272 square kilometers, and the densities in population and in medical institutions are both high. Therefore, the choice of a 3–kilometer distance between home residence and emergency room seems reasonable. In western countries, the radius of hospital service area may be set larger due to a lower population density, smaller number of hospitals, and differences in health insurance systems and coverage [34], [35].

Two issues regarding choice of modeling that merit special note are the choice of model training procedure and the use of virological surveillance. For different prediction purposes, the current Bayesian model offers short-term and long-term predictions. However, because the computation takes less than an hour for current analysis, a more intensive, say daily, updating process for the training model can be adopted, especially during influenza season, for a closer monitoring and a more prompt reaction to any detected aberration. For virological surveillance, a detected aberration may indicate higher virus activity that puts the current population at risk. Unfortunately, the schedule of laboratory work for virus isolation is usually affected by the collection practices of sentinel physicians as well as by a slowdown on weekends when only a few labs are open. For example, during Chinese New Year, the isolation rate drops sharply due to the closing of many clinics, so that the reduction in cases cannot be interpreted as a reduced risk of influenza epidemic. Likewise, any increase in isolation cases may simply be the result of cumulative cases due to holidays. From the plot of the weekly isolation rate at the stage of model validation (Figure 4(b)), it is apparent that the rise and crest of ILI visits cannot be captured by virological surveillance, while they can be detected by the proposed probabilistic measure.

There are two limitations to be overcome in the future. First, similar to other countries, the daily virological isolation rates were not available to be used as a gold standard, which leaves us unable to conduct sensitivity and specificity evaluation of this proposed method for daily ILI visits, nor to compare competing methods with true daily isolation rates. If the data can be monitored daily, then the rates in previous days would be a better explanatory variable in the model for prediction. We therefore urge the construction of a standard isolation monitoring system which will prove its significance especially during influenza pandemic. Second, the availability of numbers of historical ILI visits was limited, resulting in restricted power of the model building process. The model we use here is trained based on two-year data only, if more data had been available to train the model, the uncertainty assessment in prediction and detection could have been further improved. In addition, this model is constructed specifically for five hospitals. When data collected from other regions are to be modeled, area-specific covariates may be considered to be included in the model building process. In summary, this spatio-temporal Bayesian hierarchical model provides a measure for the probability of alert, and the freely available algorithm can serve as a complementary tool to current surveillance systems.

## Supporting Information

Figure S1 The population at risk in each buffer area of the hospital. The smallest unit indicates the distribution of Li, the five hospitals are each surrounded by a buffer area, and the color stands for the population at risk for each hospital.
(0.06 MB DOC)

Figure S2 Distributions of ILI visits and meteorological factors during 2006–2007.
(0.05 MB DOC)

Model S1 The complete model specification.
(0.02 MB DOC)

Table S1 Details of the model parameters.
(0.03 MB DOC)

Table S2 Numbers and percentages of days with posterior probabilities in different ranges.
(0.03 MB DOC)

Methods S1 WinBUGS codes for the Bayesian hierarchical model.
(0.03 MB DOC)

Methods S2 The codes of predictions and graphs in R.
(0.04 MB DOC)
