# Supplementary figures and images for: Probabilistic Daily ILI Syndromic Surveillance with a Spatio-Temporal Bayesian Hierarchical Model
Source: PLoS One. 2010 Jul 16;5(7):e11626. doi: 10.1371/journal.pone.0011626 (PMC2905374; doi:10.1371/journal.pone.0011626)

**Figure S1. The population at risk in each buffer area of the hospital.**


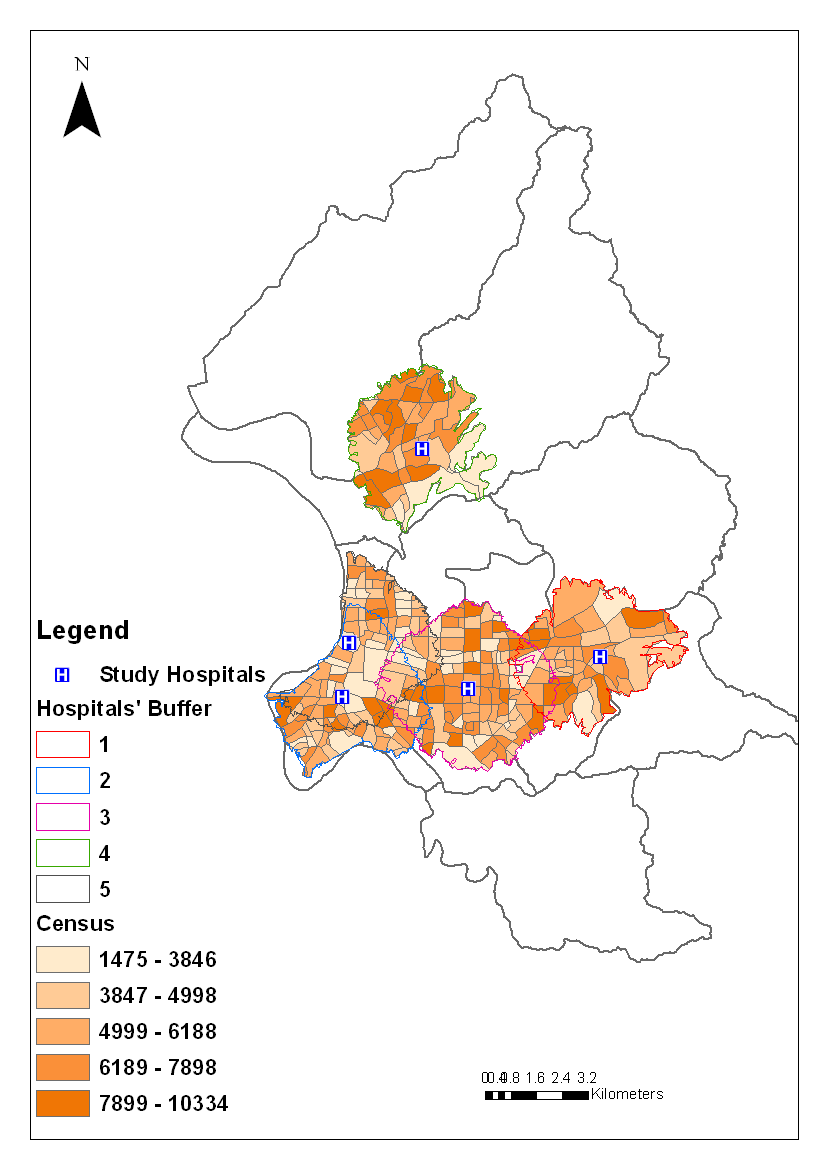

Supplement: Figure S1 — The population at risk in each buffer area of the hospital. The smallest unit indicates the distribution of Li, the five hospitals are each surrounded by a buffer area, and the color stands for the population at risk for each hospital. (0.06 MB DOC) [file pone.0011626.s001.doc]

**Figure S2. Distributions of ILI visits and meteorological factors during 2006-2007.**


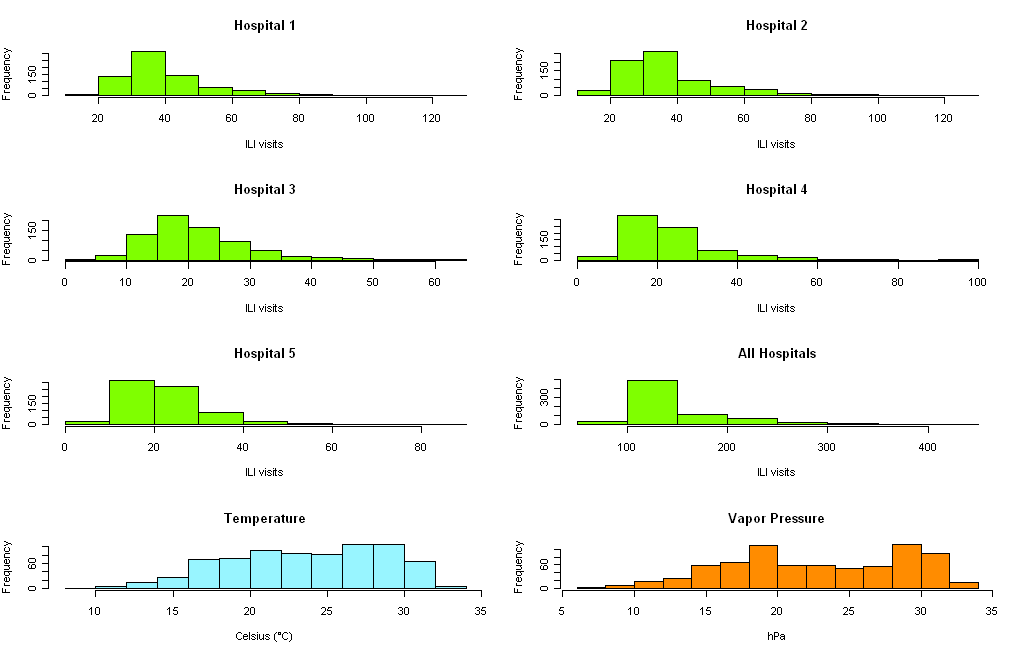

Supplement: Figure S2 — Distributions of ILI visits and meteorological factors during 2006–2007. (0.05 MB DOC) [file pone.0011626.s002.doc]
