# Supplementary material for: Probabilistic Daily ILI Syndromic Surveillance with a Spatio-Temporal Bayesian Hierarchical Model
Source: PLoS One. 2010 Jul 16;5(7):e11626. doi: 10.1371/journal.pone.0011626 (PMC2905374; doi:10.1371/journal.pone.0011626)
Supplement: Model S1 — The complete model specification. (0.02 MB DOC) [file pone.0011626.s003.doc]

**Model S1. The complete model specification**

$$\begin{matrix} y_{ji}\sim Poisson(\lambda_{ji}\times M_{i}) \\ \log(\lambda_{ji})=\alpha+U_{i}+\beta_{1}\times y_{j-1,i}+\beta_{2}\times W_{j} \end{matrix}$$

$$+\beta_{31}\times\sin\frac{Wk_{j}\times2\pi}{53}+\beta_{32}\times\cos\frac{Wk_{j}\times2\pi}{53}+\beta_{4}\times T_{j-1}+\beta_{5}\times V_{j-1}$$

$$U_{i}|U_{k}=\mu_{k},k\neq i\sim N(\frac{1}{m_{i}}\sum_{k=1}^{5} I_{ik}\mu_{k},\frac{\sigma^{2}}{m_{i}})$$

$$\alpha,\beta_{1},\beta_{2},\beta_{31},\beta_{32},\beta_{4},\beta_{5}\sim N(0,1e5)$$

$$1/\sigma^{2}\sim Gamma(0.5,0.0005)$$
