# Supplementary material for: Probabilistic Daily ILI Syndromic Surveillance with a Spatio-Temporal Bayesian Hierarchical Model
Source: PLoS One. 2010 Jul 16;5(7):e11626. doi: 10.1371/journal.pone.0011626 (PMC2905374; doi:10.1371/journal.pone.0011626)
Supplement: Table S1 — Details of the model parameters. (0.03 MB DOC) [file pone.0011626.s004.doc]

**Table S1. Details of the model parameters.**

| Parameters | Mean | SD. | 95% Credible Interval | |
| --- | --- | --- | --- | --- |
| U[1] | 0.388 | 0.009 | 0.369 | 0.404 |
| U[2] | 0.365 | 0.009 | 0.347 | 0.382 |
| U[3] | -0.766 | 0.010 | -0.785 | -0.748 |
| U[5] | 0.013 | 0.010 | -0.007 | 0.032 |
| α | -9.723 | 0.010 | -9.743 | -9.704 |
| β1 | 0.007 | 0.000 | 0.007 | 0.008 |
| β2 | 0.465 | 0.007 | 0.451 | 0.479 |
| β31 | 0.035 | 0.006 | 0.024 | 0.047 |
| β32 | 0.093 | 0.009 | 0.074 | 0.111 |
| β4 | 0.030 | 0.009 | 0.013 | 0.047 |
| β5 | -0.009 | 0.008 | -0.025 | 0.008 |
