# Supplementary material for: Probabilistic Daily ILI Syndromic Surveillance with a Spatio-Temporal Bayesian Hierarchical Model
Source: PLoS One. 2010 Jul 16;5(7):e11626. doi: 10.1371/journal.pone.0011626 (PMC2905374; doi:10.1371/journal.pone.0011626)
Supplement: Table S2 — Numbers and percentages of days with posterior probabilities in different ranges. (0.03 MB DOC) [file pone.0011626.s005.doc]

**Table S2. Numbers and percentages of days with posterior probabilities in different ranges.**

| Range for Posterior Probability | H 1 | H 2 | H 3 | H 4 | H 5 | All H |
| --- | --- | --- | --- | --- | --- | --- |
|  | No. (%) | No. (%) | No. (%) | No. (%) | No. (%) | No. (%) |
| 0.00 – 0.30 | 584(81) | 581(80) | 535(74) | 544(75) | 551(76) | 593(82) |
| 0.31 – 0.50 | 67(9) | 68(9) | 121(17) | 103(14) | 105(15) | 55(8) |
| 0.51 - 0.70 | 50(7) | 51(7) | 60(8) | 66(9) | 60(8) | 48(7) |
| 0.71 – 1.00 | 21(3) | 22(3) | 6(1) | 9(1) | 6(1) | 26(4) |
| Total | 722(100) | 722(100) | 722(100) | 722(100) | 722(100) | 722(100) |

N=722, without the first 8 days because of the temporal window of 7 days and the exclusion of the first 1 day

No.=number of days
