# Supplementary material for: Probabilistic Daily ILI Syndromic Surveillance with a Spatio-Temporal Bayesian Hierarchical Model
Source: PLoS One. 2010 Jul 16;5(7):e11626. doi: 10.1371/journal.pone.0011626 (PMC2905374; doi:10.1371/journal.pone.0011626)
Supplement: Methods S1 — WinBUGS codes for the Bayesian hierarchical model. (0.03 MB DOC) [file pone.0011626.s006.doc]

**Methods S1. WinBUGS codes for the Bayesian hierarchical model.**

**# model construction**

model

{ for (j in 2 : N1) {

# initial value is 2 for a lag of 1 day

# J: index of the day

# N1: total length of data.

for ( i in 1:N2 ){

# i: index of hospital

# N2: total number of hospitals.

ILI[j, i] ~ dpois(mu[j , i]) # set up distribution

mu[j , i]<-lamda[j , i]*pop[i]

# lamda: expected ILI occurrence rate

# pop: population at risk covered by each hospital.

log(lamda[j, i])<- (alpha + k1*ILI[j-1,i] + U[i] + k2*weekend[j] +

k31*sin((weeks[j]*2*3.14159)/53) + k32*cos((weeks[j]*2*3.14159)/53) +

k4*temp[j-1] + k7*vapor[j-1])

# log-link regression for lamda

# U: random effects following CAR model

# weekend: index variable, 1 for weekend, national holidays, and Chinese

# New Year

# week: order of the week in each year, ranging from 1 to 53

# sin: sine function and cos is the cosine function

# temp: standardized temperature, and vapor is standardized vapor pressure

} }

U[1:N2] ~ car.normal( adj[], weights[], m[], tau)

for( k in 1:sumNumNeigh ) { weights[k] <- 1 } # assign equal weight for each buffer

# followings are non-informative priors

alpha ~ dnorm(0.0, 1.0E-5)

k1 ~ dnorm(0.0, 1.0E-5)

k2 ~ dnorm(0.0, 1.0E-5)

k31 ~ dnorm(0.0, 1.0E-5)

k32 ~ dnorm(0.0, 1.0E-5)

k4 ~ dnorm(0.0, 1.0E-5)

k7 ~ dnorm(0.0, 1.0E-5)

tau ~ dgamma(0.5, 0.0005)

}

# Data

list( N1=730, N2=5,

ILI = structure( .Data = c( # past ILI data in this area

), .Dim = c(N1, N2)),

Pop = c( ), # Paste the population at risk within each hospital’s buffer

sumNumNeigh = 8,

# sum all weights. In the example, the sum is 8, 1+2+3+0+2=8

m = c(1,2,3,0,2), # number of neighbors for each hospital

adj = c(

3, # Hospital 1 has one neighbor, Hospital 3

3,5, # Hospital 2 has two neighbors, H3 and H5

1,2,5, # H3 has three neighbors

# H4 has no neighbor, an empty line is required

2,3 # H5 has two neighbors

),

weekend =c( ),

# Paste a vector of length N1 containing indexes 1 for weekends, national

# holidays and Chinese New Year, 0 for weekdays

weeks = c( ), # order of weeks, values range from 1 to 53

temp = c( ), # standardized daily average temperature

vapor = c( ) # standardized daily average vapor pressure

)

# Set up initial values for parameters

list( alpha=-0.5, k1= 0.1, U = c(0,0,0,NA,0), tau = 0.1, k2 = -40, k31 = -0.1,

k32 = -0.1, k4 = 1, k7 = 1 )
