# Supplementary material for: Probabilistic Daily ILI Syndromic Surveillance with a Spatio-Temporal Bayesian Hierarchical Model
Source: PLoS One. 2010 Jul 16;5(7):e11626. doi: 10.1371/journal.pone.0011626 (PMC2905374; doi:10.1371/journal.pone.0011626)
Supplement: Methods S2 — The codes of predictions and graphs in R. (0.04 MB DOC) [file pone.0011626.s007.doc]

**Methods S2. The codes of predictions and graphs in R**

# Designate the working directory, and place the files under this directory

setwd ('D:/R/')

# Set up the threshold based on the expected ILI visits from 7 days

# orderofmax=7 represents the highest value during last 7 days

orderofmax=7;

# Set up the prediction period, st=start time, end=end time

st=731; # e.g. the 731-th day

end=741; # e.g. the 741-th day

# Input points from WinBUGS output containing three chains

meta1=read.table("chain1.txt",sep="\t",header=F)

meta2=read.table("chain2.txt",sep="\t",header=F)

meta3=read.table("chain3.txt",sep="\t",header=F)

# Format of variables: Variable name+Chain number. Each chain has 5000 points.

# U1: WinBUGS estimation for CAR effect for Hospital 1

u11=matrix(meta1[1:5000,2],5000,1);

u21=matrix(meta2[1:5000,2],5000,1);

u31=matrix(meta3[1:5000,2],5000,1);

u1=rbind(u11,u21,u31);

# U2: WinBUGS estimation for CAR effect for Hospital 2

u12=matrix(meta1[5001:10000,2],5000,1);

u22=matrix(meta2[5001:10000,2],5000,1);

u32=matrix(meta3[5001:10000,2],5000,1);

u2=rbind(u12,u22,u32);

# U3: WinBUGS estimation for CAR effect for Hospital 3

u13=matrix(meta1[10001:15000,2],5000,1);

u23=matrix(meta2[10001:15000,2],5000,1);

u33=matrix(meta3[10001:15000,2],5000,1);

u3=rbind(u13,u23,u33);

# U5: WinBUGS estimation for CAR effect for Hospital 5

u15=matrix(meta1[15001:20000,2],5000,1);

u25=matrix(meta2[15001:20000,2],5000,1);

u35=matrix(meta3[15001:20000,2],5000,1);

u5=rbind(u15,u25,u35);

# WinBUGS estimation for alpha

alpha1=matrix(meta1[20001:25000,2],5000,1);

alpha2=matrix(meta2[20001:25000,2],5000,1);

alpha3=matrix(meta3[20001:25000,2],5000,1);

alpha=rbind(alpha1,alpha2,alpha3);

# WinBUGS estimation for k1

k11=matrix(meta1[25001:30000,2],5000,1);

k21=matrix(meta2[25001:30000,2],5000,1);

k31=matrix(meta3[25001:30000,2],5000,1);

k1=rbind(k11,k21,k31);

# WinBUGS estimation for k2

k12=matrix(meta1[30001:35000,2],5000,1);

k22=matrix(meta2[30001:35000,2],5000,1);

k32=matrix(meta3[30001:35000,2],5000,1);

k2=rbind(k12,k22,k32);

# WinBUGS estimation for k31

k131=matrix(meta1[35001:40000,2],5000,1);

k231=matrix(meta2[35001:40000,2],5000,1);

k331=matrix(meta3[35001:40000,2],5000,1);

k31=rbind(k131,k231,k331);

# WinBUGS estimation for k32

k132=matrix(meta1[40001:45000,2],5000,1);

k232=matrix(meta2[40001:45000,2],5000,1);

k332=matrix(meta3[40001:45000,2],5000,1);

k32=rbind(k132,k232,k332);

# WinBUGS estimation for k4

k14=matrix(meta1[45001:50000,2],5000,1);

k24=matrix(meta2[45001:50000,2],5000,1);

k34=matrix(meta3[45001:50000,2],5000,1);

k4=rbind(k14,k24,k34);

# WinBUGS estimation for k7

k17=matrix(meta1[50001:55000,2],5000,1);

k27=matrix(meta2[50001:55000,2],5000,1);

k37=matrix(meta3[50001:55000,2],5000,1);

k7=rbind(k17,k27,k37);

# Compute posterior mean for each parameter

b1=mean(alpha);

b12=mean(u1);

b22=mean(u2);

b32=mean(u3);

b42=0; # No neighboring effect

b52=mean(u5);

b3=mean(k1);

b4=mean(k2);

b511=mean(k31);

b512=mean(k32);

b6=mean(k4);

b8=mean(k7);

# Input observed data (X1~Xn)

LD=821; # LD: length of data

pred=read.table("predict.txt",sep="\t",header=T)

ili1=matrix(pred[1:LD,11],LD,1);

ili2=matrix(pred[1:LD,12],LD,1);

ili3=matrix(pred[1:LD,13],LD,1);

ili4=matrix(pred[1:LD,14],LD,1);

ili5=matrix(pred[1:LD,15],LD,1);

totalili=matrix(pred[1:LD,16],LD,1);

serial=matrix(pred[1:LD,1],LD,1); # the i-th day

weekend=matrix(pred[1:LD,3],LD,1)

week=matrix(pred[1:LD,4],LD,1)

z_temp=matrix(pred[1: LD,8], LD,1)

z_dewpoint=matrix(pred[1: LD,9], LD,1)

z_vapor=matrix(pred[1: LD,10], LD,1)

pop=c(284082,279622,555586,286423,266927); #population at risk with each hospital’s buffer

# Hospital 1:

x1=matrix(NA,LD ,1) ; # Create a null matrix for posterior probability

ili1a=matrix(NA,LD,1);

# Crate a null matrix for estimating log(ili1a) in Hospital 1.

# This is lamda hat 1 for estimating the exponential(ili1a) in the Hospital 1.

lamda1_hat=matrix(NA,LD,1);

meanili1=matrix(NA,LD,1); # Create a null matrix for expected ili in Hospital1

th1=matrix(NA,LD,1); # Create a null matrix for the thresholds for H1

# Estimate daily ili visits in H1

for (j in 2:LD){

ili1a[j,1]=b1+b12+b3*ili1[j - 1,1]+b4*weekend[j,1]+b511*sin((week[j,1]*2*3.14)/53)+b512*cos((week[j,1]*2*3.14)/53)+b6*z_temp[j-1,1]+b8*z_vapor[j-1,1]

lamda1_hat[j,1]=exp(ili1a[j,1])

meanili1[j,1]=lamda1_hat[j,1]*pop[1]}

# Calculate threshold and the probability exceeding threshold for H1

for (j in 8:LD) {

th=c(meanili1[j-7,1],meanili1[j-6,1],meanili1[j-5,1],meanili1[j-4,1],meanili1[j-3,1],meanili1[j-2,1],meanili1[j-1,1])

tt=sort(th)

th1[j,1]=tt[orderofmax];

x1[j,1]=(1-ppois(th1[j,1],(lamda1_hat[j,1]*pop[1])))

}

# Descriptive statistic of the probability

cat("summary probability of exceeding average",summary(x1));

# Histogram for daily probability

plot(x1~serial,type="h", main="Probability exceeding average")

# Scatter plot for the observed and predicted data

plot(meanili1~ili1,xlab="Predicted value",ylab="Observed value",main="Hospital 1",xlim=c(0,120),ylim=c(0,120))

# 45 degree diagonal line

xy=xy.coords(20:100,20:100,recycle=False)

lines(xy,col='red') # Line color=red

cor.test(ili1,meanili1) # Pearson’s correlation test

**# Repeat for Hospital 2 to Hospital 5**

#Graph for alert

st=731; # e.g., the 731-th day

end=741; # e.g., the 741-th day

interval=end-st+1; # lengeth of prediction values

op=par(mfrow=c(3,3)) # frame

threshold1=0.7 # Set up the alert lines

threshold2=0.5

threshold3=0.3

# Display daily probability for each hospital and add three color alert lines

plot(x1[st:end]~serial[st:end],type="h", main="Probability exceeding in Hospital#1")

lines(st:end,b <- rep(threshold1,times=interval),lty=1,col="red")

lines(st:end,b <- rep(threshold2,times=interval),lty=1,col="darkorange")

lines(st:end,b <- rep(threshold3,times=interval),lty=1,col="forestgreen")

plot(x2[st:end]~serial[st:end],type="h", main="Probability exceeding in Hospital#2")

lines(st:end,b <- rep(threshold1,times=interval),lty=1,col="red")

lines(st:end,b <- rep(threshold2,times=interval),lty=1,col="darkorange")

lines(st:end,b <- rep(threshold3,times=interval),lty=1,col="forestgreen")

plot(x3[st:end]~serial[st:end],type="h", main="Probability exceeding in Hospital#3")

lines(st:end,b <- rep(threshold1,times=interval),lty=1,col="red")

lines(st:end,b <- rep(threshold2,times=interval),lty=1,col="darkorange")

lines(st:end,b <- rep(threshold3,times=interval),lty=1,col="forestgreen")

plot(x4[st:end]~serial[st:end],type="h", main="Probability exceeding in Hospital#4")

lines(st:end,b <- rep(threshold1,times=interval),lty=1,col="red")

lines(st:end,b <- rep(threshold2,times=interval),lty=1,col="darkorange")

lines(st:end,b <- rep(threshold3,times=interval),lty=1,col="forestgreen")

plot(x5[st:end]~serial[st:end],type="h", main="Probability exceeding in Hospital#5")

lines(st:end,b <- rep(threshold1,times=interval),lty=1,col="red")

lines(st:end,b <- rep(threshold2,times=interval),lty=1,col="darkorange")

lines(st:end,b <- rep(threshold3,times=interval),lty=1,col="forestgreen")

# Display daily probability for total 5 hospitals and add three color alert lines

plot(totalx[st:end]~serial[st:end],type="h", main="Probability exceeding in all Hospitals")

lines(st:end,b <- rep(threshold1,times=interval),lty=1,col="red")

lines(st:end,b <- rep(threshold2,times=interval),lty=1,col="darkorange")

lines(st:end,b <- rep(threshold3,times=interval),lty=1,col="forestgreen")

par(op)
